# Analytical Determination of Heavy Metals in Water Using Carbon-Based Materials

**DOI:** 10.3390/molecules31010005

**Published:** 2025-12-19

**Authors:** Zhazira Mukatayeva, Diana Konarbay, Yrysgul Bakytkarim, Nurgul Shadin, Yerbol Tileuberdi

**Affiliations:** Department of Chemistry, Faculty of Natural Sciences and Geography, Abai Kazakh National Pedagogical University, 13, Dostyk Ave., Almaty 050010, Kazakhstan; zh.mukatayeva@abaiuniversity.edu.kz (Z.M.); nugen_87@mail.ru (N.S.); er.tileuberdi@gmail.com (Y.T.)

**Keywords:** carbon-based sensors, heavy metals, on-site electrochemical detection, self-powered Hg^2+^ sensor, Mxene, DPV

## Abstract

This review presents a critical and comparative analysis of carbon-based electrochemical sensing platforms for the determination of heavy metal ions in water, with emphasis on Pb^2+^, Cd^2+^, and Hg^2+^. The growing discharge of industrial and mining effluents has led to persistent contamination of aquatic environments by toxic metals, creating an urgent need for sensitive, rapid, and field-deployable analytical technologies. Carbon-based nanomaterials, including graphene, carbon nanotubes (CNTs), and MXene, have emerged as key functional components in modern electrochemical sensors due to their high electrical conductivity, large surface area, and tunable surface chemistry. Based on reported studies, typical detection limits for Pb^2+^ and Cd^2+^ using differential pulse voltammetry (DPV) on glassy carbon and thin-film electrodes are in the range of 0.4–1.2 µg/L. For integrated thin-film sensing systems, limits of detection of 0.8–1.2 µg/L are commonly achieved. MXene-based platforms further enhance sensitivity and enable Hg^2+^ detection with linear response ranges typically between 1 and 5 µg/L, accompanied by clear electrochemical or optical signals. Beyond conventional electrochemical detection, this review specifically highlights self-sustaining visual sensors based on MXene integrated with enzyme-driven bioelectrochemical systems, such as glucose oxidase (GOD) and Prussian blue (PB) assembled on ITO substrates. These systems convert chemical energy into measurable colorimetric signals without external power sources, enabling direct visual identification of Hg^2+^ ions. Under optimized conditions (e.g., 5 mg/mL GOD and 5 mM glucose), stable and distinguishable color responses are achieved for rapid on-site monitoring. Overall, this review not only summarizes current performance benchmarks of carbon-based sensors but also identifies key challenges, including long-term stability, selectivity under multi-ion interference, and large-scale device integration, while outlining future directions toward portable multisensor water-quality monitoring systems.

## 1. Introduction

Heavy metal contamination of water resources has become a critical global environmental problem due to the persistence, bioaccumulation, and high toxicity of these pollutants [1,2,3,4,5,6,7]. Industrial discharge, mining activities, domestic wastewater, and agricultural runoff continuously introduce toxic metal ions into aquatic environments, leading to long-term ecological instability and serious risks to human health [5,6]. Even at trace concentrations, heavy metals can induce oxidative stress, DNA damage, metabolic disorders, and irreversible organ dysfunction [7,8,9,10,11,12]. In this review, the term “heavy metals” specifically refers to Pb, Cd, and Hg, which are among the most hazardous and environmentally relevant toxic elements.

Recent toxicological studies further demonstrate that exposure to heavy metals induces a wide range of molecular, genetic, and epigenetic disturbances in humans and animals. Chromium(VI), arsenic, cadmium, and mercury have been shown to trigger DNA damage, chromosomal instability, oxidative stress, and disrupted immune responses, contributing to increased carcinogenic risk and metabolic disorders [13,14,15,16,17,18,19,20]. Long-term exposure even at low concentrations may alter antioxidant defense systems, impair renal and hepatic function, and interfere with neurodevelopment, particularly in vulnerable populations such as children and adolescents [21,22,23,24]. Moreover, chromium and arsenic compounds exhibit complex mechanisms of toxicity involving DNA adduct formation, aberrant methylation patterns, and protein damage, further exacerbating their hazardous impact on human health [25,26,27,28]. Together, these findings highlight the urgent need for sensitive, accurate, and rapid detection strategies to monitor heavy metal contamination in environmental water sources.

Reliable detection of heavy metals in water is essential for environmental monitoring, pollution control, and public health protection. Numerous analytical techniques have been developed for this purpose, including spectroscopic methods such as atomic spectroscopy and X-ray fluorescence (XRF) [29,30,31,32,33,34,35,36], chromatographic techniques such as high-performance liquid chromatography (HPLC) and ion chromatography (IC) [37,38], mass spectrometry-based methods such as inductively coupled plasma mass spectrometry (ICP-MS) [39], and optical methods including colorimetry, ultraviolet–visible spectroscopy (UV–Vis), and chemiluminescence (CL) [40,41,42]. Although these laboratory-based techniques offer excellent sensitivity and selectivity, they require expensive instrumentation, well-controlled laboratory environments, trained personnel, and complex sample pretreatment. As a result, they are poorly suited for rapid, low-cost, real-time, and on-site monitoring of heavy metals in field conditions.

**Figure 1:**
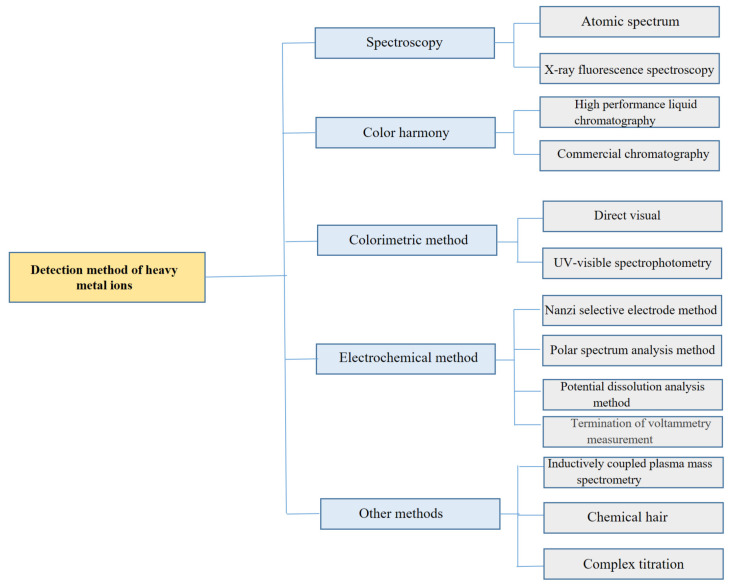
Main Methods for Detecting Heavy Metals in Water Resources.

Currently, the following methods are used to detect heavy metal ions in water resources (as shown in Figure 1):

Electrochemical detection techniques have emerged as highly attractive alternatives due to their inherent advantages, including high sensitivity, fast response, low cost, simple instrumentation, and excellent compatibility with portable and miniaturized devices [43]. Common electrochemical approaches for heavy metal detection include ion-selective electrodes (ISEs) [44,45,46,47], anodic stripping voltammetry (ASV) [48,49], polarographic analysis [50], and potentiometric stripping analysis (PSA) [51]. Despite their strong analytical performance, practical electrochemical sensing still faces several challenges, such as electrode surface fouling, limited reproducibility, matrix interference in complex water samples, long-term electrode stability, and the demand for low-power, field-deployable operation.

To overcome these limitations, carbon-based materials have been extensively explored as advanced electrode modifiers and sensing platforms. Materials such as graphene, multi-walled carbon nanotubes, and MXene exhibit outstanding electrical conductivity, large specific surface area, chemical stability, and tunable surface functionalization. These properties significantly enhance electron-transfer kinetics, adsorption capacity toward metal ions, and overall sensor sensitivity. Consequently, carbon-based electrochemical sensors offer a powerful strategy for achieving sensitive, stable, and portable detection of heavy metal ions in water. Furthermore, the rapid development of self-powered and visual sensing platforms based on carbon materials, particularly MXene-assisted bioelectrochemical systems, has opened new opportunities for in situ and on-site environmental monitoring without the need for external power sources. These systems integrate electrochemical energy conversion with optical signal output, enabling direct, rapid, and user-friendly detection of toxic metal ions.

Table 1 provides a summary of LOD and MDL values for Pb^2+^, Cd^2+^ and Hg^2+^ obtained using the developed electrochemical sensing platforms. LOD values were calculated using the standard equation LOD = 3σ/S, while MDL values were determined following the EPA statistical protocol. The obtained limits are significantly below WHO and EPA regulatory thresholds, confirming that the sensors are applicable for early-stage detection of heavy metal ions in water.

Table 2 provides a comparison of the analytical performance of the developed sensors with selected literature reports. The table summarizes electrode types, detection techniques, target metal ions, and corresponding LOD values. The results demonstrate that the bare GCE provides competitive sensitivity even without surface modification, while the MXene-based self-powered platform enables Hg^2+^ detection within environmentally relevant concentration ranges.

Table 3 presents key electrochemical performance parameters of the developed sensors, including linear range, sensitivity, repeatability (RSD%), and operational stability. The low RSD values (≤7%) indicate good repeatability, while stable sensor responses confirm suitability for portable and field-applicable monitoring of Pb^2+^, Cd^2+^ and Hg^2+^ ions.

In this review, we systematically summarize recent advances in carbon-based electrochemical sensors for the determination of Pb^2+^, Cd^2+^, and Hg^2+^ in water. Key detection mechanisms, analytical performance, and practical limitations are critically discussed. Special attention is given to portable, self-powered, and visual sensing systems, highlighting their potential for future field-applicable water monitoring technologies.

## 2. Development of Electrochemical Systems Based on Carbon Materials for the Detection of Heavy Metals

### 2.1. Conventional Carbon Nanomaterials

Carbon nanomaterials are widely used in the development of electrochemical sensors for the detection of heavy metals due to their excellent electrical conductivity, large specific surface area, and strong adsorption ability. Carbon nanotubes (CNTs) are generally classified into single-walled carbon nanotubes (SWCNTs) and multi-walled carbon nanotubes (MWCNTs). Compared to sp^3^ hybridization, the sp^2^ hybridization of carbon atoms provides CNTs with high tensile strength and outstanding electrical properties. The π-bonding formed by the overlap of p-electron orbitals enables fast electron transfer, while the large surface area enhances catalytic activity. These features make CNTs highly suitable for electrochemical detection of toxic heavy metals [52].

From a sensing mechanism perspective, the detection of heavy metal ions on carbon-based electrodes is governed by surface adsorption, electrostatic interaction, and redox reactions at the electrode–solution interface. Functional groups on carbon nanomaterials facilitate the accumulation of Pb^2+^, Cd^2+^, and Hg^2+^ ions during the preconcentration step. Subsequent anodic stripping produces distinct current peaks, enabling sensitive quantitative analysis.

Preconcentration of analytes on the electrode surface remains a key strategy for achieving low detection limits in portable electrochemical systems. EDTA-functionalized polymer–carbon nanocomposites, MOF-integrated carbon electrodes, and magnetic sorbent-assisted platforms have been shown to effectively capture Pb^2+^ and Cd^2+^ ions through coordination and adsorption interactions, resulting in improved stripping efficiency, lower detection limits, and enhanced repeatability [53,54,55,56,57].

Matlou et al. [58] reported that SWCNTs modified with polyaminobenzene sulfonic acid were successfully applied for the detection of Hg^2+^ in water using SWASV. The modified electrode showed improved sensitivity and a low LOD of 0.06 μM.

Wang et al. [59] reported that functionalized MWCNTs were employed as electrode modifiers for Pb^2+^ detection using DPASV. A good linear relationship between peak current and metal ion concentration was observed, with a LOD of 0.01 μM. These studies confirm that CNT-based electrochemical sensors exhibit high sensitivity and low detection limits for heavy metal ions.

**Figure 2:**
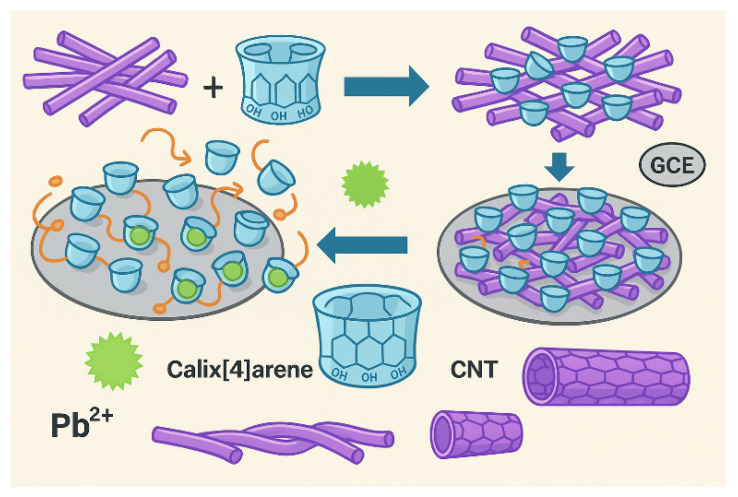
Schematic illustration of the preparation and electroanalysis mechanism of the TCA/MWCNT-modified electrode [59].

Figure 2 illustrates the preparation process of the modified electrode and the mechanism of electroanalysis.

Graphene is a two-dimensional carbon material with exceptional electrical conductivity and mechanical strength, making it an ideal material for electrochemical sensor design. Owing to its large surface area, graphene facilitates high loading of active sites and rapid electron transfer. Therefore, graphene and its derivatives such as graphene oxide (GO), reduced graphene oxide (rGO), and graphene quantum dots (GQDs) are widely used in electrochemical sensing systems [60].

Guan Huanan et al. [61] developed a graphene-modified glassy carbon electrode for the detection of Hg^2+^ and Cu^2+^, achieving high sensitivity and low detection limits. Chen Xi et al. [62] synthesized nitrogen-doped graphene for heavy metal detection and reported a LOD of 2.0 nM for Cd^2+^ and 0.1 nM for Pb^2+^. Priya et al. [63] applied a porous rGO/AuNP composite for simultaneous detection of Pb^2+^ and Cd^2+^, achieving detection limits of 2 ng/L and 3 ng/L, respectively.

**Figure 3:**
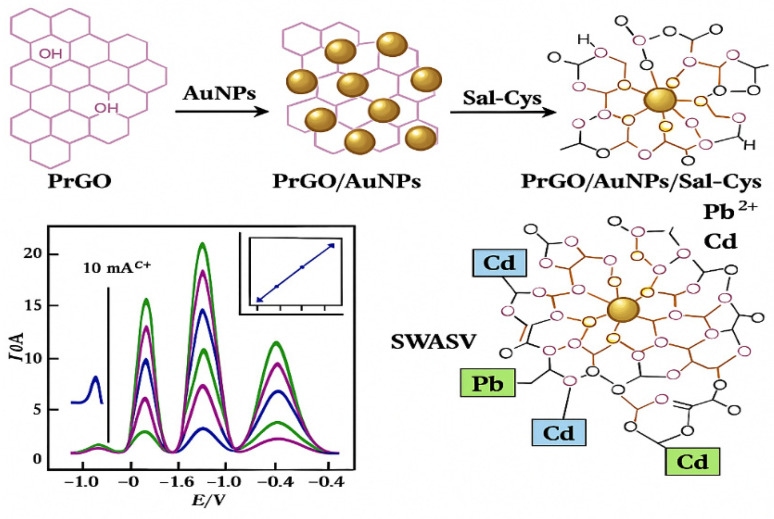
Schematic illustration of the interaction between Cd^2+^ and Pb^2+^ with the surface of the PrGO/AuNPs/Sal-Cys/GCE [64].

Xu He et al. [64] synthesized graphene quantum dots and developed a modified GCE, with LODs of 16.1 nM for Pb^2+^ and 35.7 nM for Cd^2+^. These results demonstrate the high electrochemical activity of graphene-based materials in trace heavy metal detection (Figure 2). Furthermore, the interaction mechanism between Cd^2+^/Pb^2+^ ions and the PrGO/AuNPs/Sal-Cys-modified electrode is illustrated in Figure 3, which provides a clearer understanding of the adsorption and electrochemical stripping behavior on graphene-based hybrid surfaces.

C60 is an important carbon isomer composed entirely of *sp*^2^-hybridized carbon atoms. C60 tends to accept electrons and, under certain conditions, donate them, exhibiting rich electrochemical properties [65]. Like other carbon nanomaterials, C60 is widely used as an electrode material. Han et al. [66] developed an ASV-based method for the detection of heavy metal ions using C60. They utilized C60 as an electrode modifier and prepared a novel, highly sensitive modified electrode (C60-chitosan/GCE) for electrochemical detection of heavy metal ions based on C60 and chitosan. This method features a low detection limit, good repeatability, and excellent performance for simultaneous detection of copper, mercury, lead, and cadmium.

### 2.2. Carbon Materials Derived from MOFs

New metal–organic frameworks (MOFs), consisting of metal ions and multifunctional organic ligands, possess several advantages such as high BET-specific surface area, rich metal–organic content, large pore volume, and the ability to tune their structure and composition. These features make them promising self-sacrificing templates and precursors for the synthesis of various carbon nanomaterials [67]. Compared to other carbon-based catalysts, carbon nanomaterials derived from MOFs can serve as direct catalysts or catalyst supports for many important reactions due to their tunable morphology, high layered porosity, and ease of functionalization with other dopant atoms or metals/metal oxides [68,69]. Tan [70] and co-authors combined an amino-functionalized zirconium-based MOF (NH_2_-UiO-66) with a zeolitic imidazolate framework based on zinc (ZIF-8) to develop a core–shell hybrid material (NH_2_-UiO-66@ZIF-8, NU66@Z8). The NU66@Z8, combined with carboxylated multi-walled carbon nanotubes (CMWCNTs), was deposited on a GCE to fabricate an electrochemical sensor for the detection of lead and copper ions. Under optimized conditions, the detection limit for lead using this sensor was 1 nM.

### 2.3. Biomass-Based Carbon Materials

Compared with conventional fossil-derived carbon materials, biomass-derived carbon materials exhibit abundant surface functional groups, hierarchical porosity, and low production cost [71]. Such materials are typically prepared through carbonization and activation of agricultural waste and plant residues.

**Figure 4:**
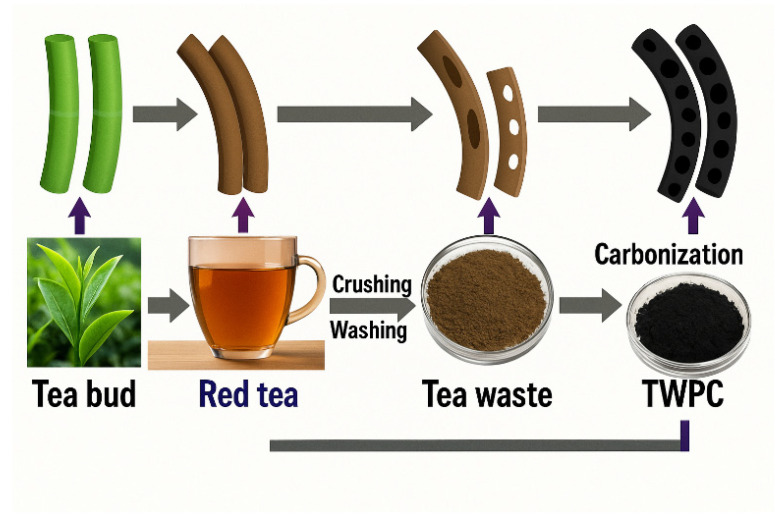
Porous biomass-based material synthesized from tea waste using a combination of carbonization and activation [75].

The use of biomass materials not only helps to effectively address the complex production process of traditional carbon materials but also reduces waste, which holds significant practical importance for energy conservation, emission reduction, and environmental protection. Biomaterials are mainly converted into porous carbon materials through a combination of carbonization and chemical activation. Impurities in the biomolecular framework can be removed from the biomass through high-temperature carbonization in an inert gas atmosphere, resulting in the formation of a porous carbon skeleton [72,73]. Due to the wide variety of biomass sources, diverse biomaterials such as persimmon branches [74], tea waste [75], corn cobs [76], cork oak [77], eggplants [78], palm shells [79], etc., can be used to prepare biomass-based porous carbon materials. Figure 4 shows a carbon-based biomaterial synthesized from tea waste using a simple method that combines carbonization and activation [80]. Zhang et al. [81] reported a biomass-derived porous carbon prepared from grapefruit peel, which showed excellent electrochemical performance for the detection of Pb^2+^, Cd^2+^, and Cu^2+^ using SWASV. The sensor exhibited high sensitivity, good repeatability, and low detection limits. These results indicate that biomass-based carbon materials are promising sustainable alternatives for electrochemical heavy metal detection.

### 2.4. Electrochemical Sensing Mechanisms of Carbon-Based Materials

The electrochemical detection of heavy metal ions on carbon-based electrodes is fundamentally governed by a combination of surface adsorption, electrostatic interaction, interfacial charge transfer, and redox reactions occurring at the electrode–electrolyte interface. At the molecular level, Pb^2+^, Cd^2+^, and Hg^2+^ ions are initially transported toward the electrode surface by diffusion and electrostatic attraction. The presence of oxygen-containing functional groups such as –COOH, –OH, and –C=O on carbon nanomaterials enables strong coordination interactions with metal ions, promoting their stable preconcentration on the surface.

During anodic stripping voltammetry (ASV), the electrochemical sensing process consists of two main steps: (i) a deposition step, in which metal ions are electrochemically reduced and accumulated in their metallic state on the electrode surface; and (ii) a stripping step, in which the deposited metals are re-oxidized, producing well-defined current peaks that are directly proportional to the metal ion concentration. This preconcentration–stripping mechanism leads to significant signal amplification and enables ultra-trace detection of heavy metal ions. The π-electron-conjugated structure of carbon nanotubes and graphene facilitates rapid electron transfer across the electrode surface, significantly reducing charge-transfer resistance. In addition, the large specific surface area and abundant defect sites provide numerous active adsorption centers, thereby enhancing both sensitivity and detection stability. When bismuth or antimony films are introduced, intermetallic alloy formation improves nucleation efficiency and suppresses hydrogen evolution, resulting in higher signal-to-noise ratios and improved analytical performance.

At the molecular scale, the electrochemical signal originates from the redox conversion of surface-bound metal species. The adsorption–diffusion–reduction–oxidation sequence constitutes the core sensing mechanism that governs the analytical response of carbon-based electrochemical sensors. These principles are consistent with recent comprehensive reviews showing that carbon-based electrochemical sensors, including graphene and carbon nanotube platforms, achieve low detection limits and high selectivity for Pb^2+^, Cd^2+^, and Hg^2+^ through surface functionalization, preconcentration effects, and efficient electron-transfer pathways [82].

The application of bismuth film electrodes has been widely reported as an environmentally friendly alternative to mercury-based electrodes for the simultaneous determination of Pb^2+^ and Cd^2+^. Wang et al. [83] demonstrated that in situ plated bismuth films on glassy carbon electrodes significantly enhance stripping signal intensity due to the formation of fusible Bi–metal alloys and improved mass transport at the electrode surface.

Recent studies have demonstrated that metal–organic framework (MOF)–based electrochemical sensors provide high sensitivity and selectivity for the simultaneous detection of Pb^2+^, Cd^2+^, and Hg^2+^ ions, owing to strong metal–ligand interactions and efficient charge-transfer pathways, making them highly suitable for stripping voltammetric analysis of toxic heavy metals [84].

Recent advances in carbon-based electrochemical sensors have significantly expanded their applicability for on-site monitoring of heavy metals in water and food samples. Graphene- and carbon nanotube-based hybrid electrodes provide high surface area, fast electron transfer, and enhanced sensitivity, enabling simultaneous detection of Pb^2+^, Cd^2+^, and Hg^2+^ at trace levels [85,86,87,88]. In particular, hybrid nanostructures combining graphene derivatives with metal oxides or conducting polymers have demonstrated improved charge-transfer kinetics and mechanical stability, which are critical for portable and field-deployable sensing platforms [89,90,91]. Furthermore, nanocomposite strategies integrating chelating agents and redox-active modifiers have been reported to improve selectivity and signal stability in complex environmental matrices [92,93,94].

## 3. Development of Electrochemical Sensors Based on MXene Materials

**Figure 5:**
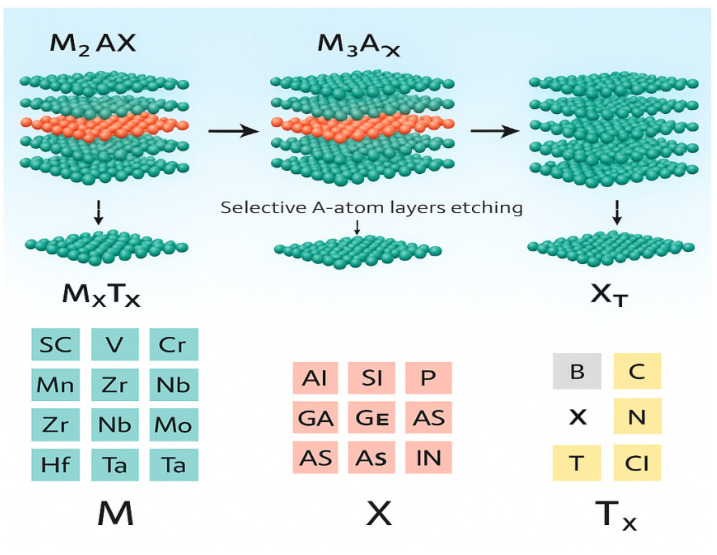
Schematic representation of the MAX phase structure with *n* = 1–4 and the structure of MXene, showing transition metals, carbon/nitrogen, A-group elements, and etched surface terminations [105].

MXenes are a class of two-dimensional transition metal carbides and nitrides and cannot be strictly classified as carbon-based materials. The general structure of MAX phases and the formation of MXene layers through selective etching are illustrated in Figure 5. The commonly used etching routes for producing MXenes from MAX phases—including fluoride-based, alkaline, hydrothermal, and Lewis acid etching—are summarized in Table 4. However, due to their outstanding electrical conductivity, hydrophilicity, and strong interaction with carbon electrodes, MXenes are frequently integrated into carbon-based electrochemical sensing platforms. Therefore, MXene-based systems are discussed in this review as carbon-related hybrid electrochemical sensors.

MXenes, particularly Ti_3_C_2_T_x_, have attracted increasing attention as carbon-related hybrid platforms due to their metallic conductivity, hydrophilic surface terminations, and strong interfacial compatibility with carbon electrodes. Well-established synthesis and processing protocols enable the fabrication of stable MXene films with tunable surface chemistry, which is advantageous for electrochemical sensing applications [95]. Recent studies have demonstrated that MXene-based electrodes enable simultaneous determination of Cd^2+^ and Pb^2+^ with high sensitivity and wide linear ranges, supporting their use in portable electrochemical detection systems [96,97,98].

Although MXenes are not carbon-based materials, they are frequently integrated with carbon electrodes and electrochemical sensing platforms because of their excellent electrical conductivity and strong affinity toward metal ions. Their hydrophilic surface and abundant functional groups provide numerous active sites for metal ion adsorption and facilitate fast electron transfer during electrochemical reactions, thereby significantly enhancing sensor sensitivity and signal stability [99,100,101,102,103,104].

The electrochemical detection of heavy metal ions on MXene-modified electrodes is primarily governed by surface adsorption, electrostatic interaction, and redox reactions at the MXene–electrolyte interface. During anodic stripping voltammetry, metal ions are first preconcentrated on the MXene-modified surface through electrostatic attraction and coordination with surface functional groups. Subsequently, the accumulated metal ions undergo electrochemical oxidation, generating well-defined stripping peaks. This preconcentration–stripping process leads to significant signal amplification and enables ultra-trace detection of Pb^2+^, Cd^2+^, and Hg^2+^ ions.

In recent years, various MXene-based electrochemical sensors have been developed for the sensitive determination of Pb^2+^ and Cd^2+^. He et al. [105] fabricated a BiNPs/Ti_3_C_2_T_x_ composite electrode for the simultaneous detection of Pb^2+^ and Cd^2+^ using anodic stripping voltammetry. The sensor exhibited excellent analytical performance with detection limits of 10.8 nM for Pb^2+^ and 12.4 nM for Cd^2+^, demonstrating the strong potential of MXene-based materials in trace heavy metal detection. Other studies have also shown that the integration of MXenes with metal nanoparticles, polymers, and carbon nanomaterials further enhances adsorption capacity, selectivity, and long-term stability [106,107,108,109,110].

Beyond conventional electrochemical sensing, MXenes have recently been applied in the development of self-powered electrochemical and visual sensing platforms for Hg^2+^ detection. In such systems, MXenes function as highly conductive electrode materials that enable efficient electron transport and signal generation without the need for an external power source. In our study, a MXene-based self-powered visual electrochemical system was developed for Hg^2+^ detection by integrating MXene with an enzyme-driven bioelectrochemical process. Owing to the high conductivity and surface activity of MXene, the system exhibits distinct visual and electrochemical responses toward Hg^2+^ ions, providing a rapid, portable, and energy-efficient strategy for on-site monitoring of mercury contamination in water.

**Table 4:** Various methods for MXene etching.

Etching Method	Etching Agent	Etching Temperature (°C)	Reference
Fluoride Acids	HF	Room-55	[105]
	H_2_O_2_ + HF	40	[106]
	HCl + HF	35–55	[107]
	HCl + (Na, K, or NH_4_F)	30–60	[108]
	NH_4_HF_2_	Room	[109]
Alkaline Methods	NaOH	270	[110]
Hydrothermal Method	NaBF_4_, HCl	180	[111]
Molten Salts	LiF + NaF + KF	550	[112]
Electrochemical	NH_4_Cl/TMAOH	Room	[113]
Lewis Acids	ZnCl_2_	550	[114]
Chemical Vapor Phase	3D Graphene/Ti_3_AlC_2_/PDMS membrane	-	[115]

Beyond laboratory-based sensing platforms, MXene-assisted electrochemical systems have been successfully integrated into portable and smartphone-enabled devices, highlighting their strong potential for decentralized and on-site environmental monitoring. The combination of MXenes with miniaturized electrochemical readout units enables rapid, low-power, and field-deployable detection of heavy metals in water and food matrices, which is essential for real-time contamination assessment [111].

Despite these advantages, certain challenges associated with MXene-based electrochemical sensors still remain. MXenes are susceptible to surface oxidation and structural degradation under long-term aqueous exposure, which may affect their electrochemical stability and sensing performance. Therefore, future research should focus on improving MXene stability through surface modification, composite construction, and device-level integration. With continued development, MXene-based platforms are expected to play an increasingly important role in portable, self-powered, and in situ detection of toxic heavy metal ions.

## 4. Objective and Scope of the Research

Pollution of water bodies poses a threat to the use of water resources. Heavy metals in water are difficult to remove naturally, and most of them accumulate in aquatic environments, leading to concentrations that exceed permissible limits and render the water unsuitable for use.

To achieve a more scientific understanding of the state of the aquatic environment and to utilize water resources more effectively, it is important to focus on analyzing the content of heavy metals in water bodies. Conventional analysis of heavy metal ions depends on specialized and strict laboratory conditions, which presents significant limitations. In contrast, the electrochemical method, as an important trace analysis technique, is simple to operate and can be miniaturized, offering significant advantages for rapid analysis of heavy metals. Research into electrochemical sensors, devices, and analytical methods enables convenient, fast, and accurate quantitative determination of heavy metal content in water, which is of great importance for residential environments, food safety, and both industrial and agricultural production.

To enable faster, more sensitive, and more accurate detection of common heavy metals in water such as Pb^2+^, Cd^2+^, and Hg^2+^, as well as to gain a more scientific understanding of the aquatic environment and improve the use of water resources, the following research will be carried out in this study: At present, many literature sources describe methods for the detection of Pb^2+^ and Cd^2+^ in water using various electrode modification techniques. These methods involve relatively complex electrode modification processes and have certain limitations in terms of operational simplicity. In this study, to achieve simpler and faster detection of Pb^2+^ and Cd^2+^, a three-electrode system was developed using a glassy carbon electrode (GCE) as the working electrode, silver/silver chloride (Ag/AgCl) as the reference electrode, and a platinum wire as the counter electrode to detect Pb^2+^ and Cd^2+^ in water. The limits of detection (LODs) and method detection limits (MDLs) for both elements will be determined, and sensitive detection of Pb^2+^ and Cd^2+^ in water using unmodified GCE will be demonstrated.

In Study 1, the enrichment of heavy metals was performed by stirring. In the present study, a laboratory-developed integrated device for the detection of heavy metal ions (a thin-layer cell) was used, in which stirring was replaced by flow-based enrichment. A glassy carbon electrode (GCE) was used as the working electrode, Ag/AgCl as the reference electrode, and a platinum wire as the counter electrode. Detection of Pb^2+^ and Cd^2+^ in water was carried out, and the limits of detection (LODs) and method detection limits (MDLs) for both elements were determined, enabling sensitive detection of Pb^2+^ and Cd^2+^ in water using the thin-layer cell system.

In Studies 1 and 2, electrochemical detection of Pb^2+^ and Cd^2+^ in water was conducted. Since bare GCE is not suitable for the detection of mercury, which is commonly found in water, a new electrochemical method based on carbon materials was developed in this work for the sensitive detection of Hg^2+^ in water. A two-dimensional transition metal carbide (MXene) was used as the non-enzymatic anode, and glucose oxidase (GOD)/Prussian Blue (PB) was used as the biocathode. Both components were integrated into an indium tin oxide (ITO) substrate, forming a self-powered visual sensor for the detection of Hg^2+^, enabling sensitive detection of Hg^2+^ in water.

Compared to traditional sensors, self-powered electrochemical sensors (SPES) do not require batteries or external power sources, allowing for rapid on-site testing.

## 5. Experimental Section

### 5.1. Experimental Methods and Principles

**Figure 6:**
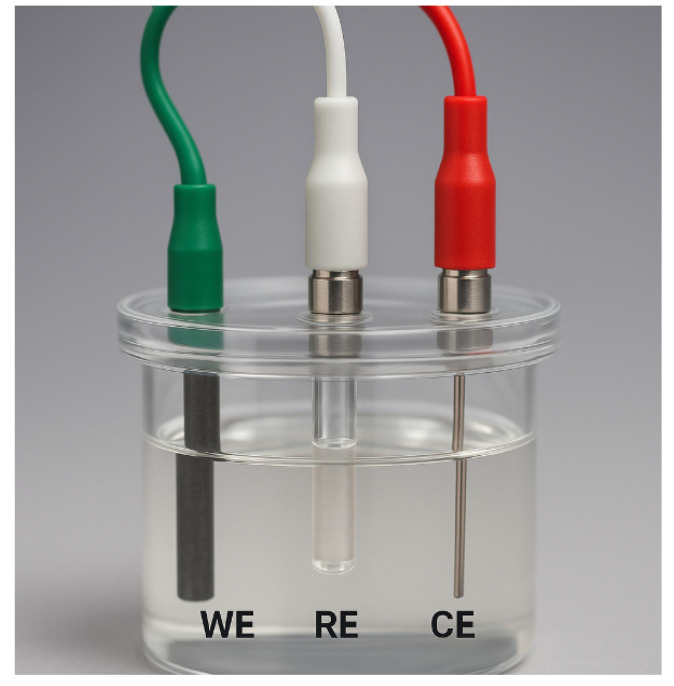
Schematic diagram of the heavy metal ion detection setup using a three-electrode system.

A three-electrode electrochemical system was used in the experiment. The three-electrode system is the most commonly used setup in electrochemistry. Its configuration is shown in Figure 6 and includes a working electrode, a reference electrode, and a counter electrode. In this experiment, a glassy carbon electrode (GCE) was used as the working electrode, a silver/silver chloride (Ag/AgCl) electrode served as the reference electrode, and a platinum wire was used as the counter electrode. The three electrodes were connected to the corresponding terminals of the electrochemical workstation. Using differential pulse voltammetry (DPV), Pb^2+^ and Cd^2+^ ions in the aqueous phase, composed of a supporting electrolyte solution, were detected.

**Figure 7:**
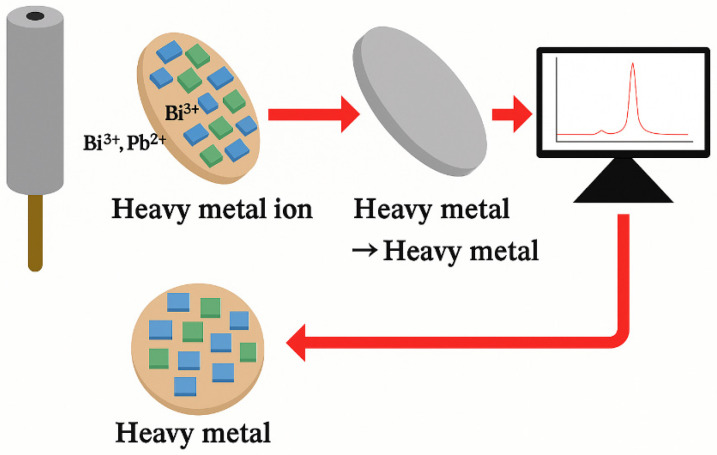
Schematic diagram of heavy metal ion detection in water using DPV (Differential Pulse Voltammetry).

DPV (Differential Pulse Voltammetry) is a powerful electrochemical analytical technique used to detect and quantify various metal ions at extremely low concentrations in aqueous media. It allows for the identification and quantification of electroactive substances in solutions at nanomolar concentrations or lower, offering sensitivity comparable to technologies such as inductively coupled plasma mass spectrometry (ICP-MS). The detection of heavy metal ions using DPV typically involves two main stages (see Figure 7). First, the heavy metal ions undergo constant potential preconcentration under stirring conditions, allowing the target substance to accumulate and concentrate on the working electrode. Then, the potential of the working electrode is swept from a negative to a positive value, causing the preconcentrated substance on the electrode surface to re-dissolve into the solution [112]. During the re-dissolution of each electro-enriched substance, a stripping peak is generated. The peak potential of each signal can be used to identify the oxidized substance, while the peak current is related to the concentration of the substance in the solution [113]. As illustrated in Figure 7, the colored symbols represent accumulated heavy metal ions on the electrode surface, where the different colors are used only to distinguish ions visually.

The bismuth film electrode is a highly attractive type of electrode due to its high sensitivity, low toxicity (compared to mercury electrodes), wide cathodic potential range, and its ability to deposit on various surfaces (such as gold, platinum, glassy carbon, carbon paste, and carbon fiber). The bismuth film can form alloys and intermetallic compounds with certain metals and metalloids such as cadmium, lead, antimony, thallium, gallium, indium, and others. The formation of such alloys promotes the nucleation process during the deposition of heavy metals, enabling effective enrichment of heavy metals and thus increasing analytical sensitivity [114]. Moreover, it is insensitive to oxygen interference and exhibits better peak separation performance compared to mercury film electrodes. In 2000, Wang [83] and colleagues first used a GCE as the working electrode, Ag/AgCl as the reference electrode, and a platinum wire as the counter electrode, developing a bismuth film-modified GCE for the detection of metal ions. During the stripping of metal ions, distinct and well-separated stripping peaks were observed. The measured stripping potentials were approximately −0.20 V for Bi^3+^, −0.58 V for Pb^2+^, and −0.79 V for Cd^2+^. Based on the excellent performance of bismuth films in detecting heavy metal ions [115,116,117,118,119], this study utilized a bismuth film coated on a glassy carbon electrode for the detection of Pb^2+^ and Cd^2+^.

During the DPV detection process of Bi^3+^ on the glassy carbon electrode, a cycle of accumulation and stripping takes place. After this, it is necessary to clean the surface of the GCE at a specific potential to activate or renew it. To remove the bismuth film from the electrode surface, a potential higher than the oxidation potential of Bi^3+^ must be selected—typically in the range of 0.0–0.3 V—and applied for 30 s to effectively clean the surface [120,121]. Electrochemical cleaning allows for the rapid renewal of the electrode surface, ensuring good stability and reproducibility of the working electrode.

### 5.2. Preparation of Required Solutions

(1) GCE Test Solution: The GCE test solution is a mixture of aqueous solutions containing 5 mM K_3_Fe(CN)_6_, 5 mM K_4_Fe(CN)_6_, and 0.1 M KCl. Accurately weigh 0.164 g of solid K_3_Fe(CN)_6_, 0.211 g of solid K_4_Fe(CN)_6_, and 0.74 g of solid KCl. Place them in a beaker and add a small amount of deionized water to fully dissolve and mix the three solids. Then, transfer the mixture to a 100 mL volumetric flask and add deionized water to reach the final volume of 100 mL.
(2) Sample Test Solution: Transfer 1 mL of the Bi^3+^ standard solution with a concentration of 100 μg/mL from the stock bottle into a 10 mL centrifuge tube for further use. Then, pipette 1 mL each of the Pb^2+^ and Cd^2+^ standard solutions with a concentration of 1000 μg/mL and dilute them with distilled water to a concentration of 100 μg/mL to obtain stock solutions of Pb^2+^ and Cd^2+^. During testing, dilute the 100 μg/mL Pb^2+^ and Cd^2+^ stock solutions to a working concentration of 2 μg/mL.
(3) Acetate–Sodium Acetate Buffer (pH = 4.5): Since Bi^3+^ easily undergoes hydrolysis under neutral or alkaline conditions to form BiOCl precipitate, which can interfere with the testing process, hydrolysis is suppressed in acidic media. Therefore, a commonly used laboratory buffer solution—0.1 M acetic acid–sodium acetate buffer with pH = 4.5—was selected as the supporting electrolyte for this experiment. Accurately weigh 3.86 g of glacial acetic acid and 2.93 g of sodium acetate, place them in a beaker, add a small amount of deionized water to fully dissolve and mix, then transfer the solution to a 1 L volumetric flask and make up the volume to 1 L with deionized water. The pH of the solution was measured using a pH meter and confirmed to be 4.5. After preparation, store all solutions in a refrigerator at 4 °C.

### 5.3. Pre-Treatment of GCE (Glassy Carbon Electrode)

When conducting experiments using GCE, it is essential to carry out a pre-treatment procedure to enhance its electrochemical performance [122]. This pre-treatment generally involves polishing the electrode with alumina (Al_2_O_3_) powders of various particle sizes, followed by ultrasonic cleaning of the GCE surface. The purpose of the pre-treatment is to expose fresh carbon atoms on the electrode surface, which act as active sites for electrochemical reactions [123,124]. After polishing, it is necessary to verify whether the peak potential difference (ΔΦ) observed in the cyclic voltammetry (CV) curve—recorded in a standard reversible system consisting of K_3_Fe(CN)_6_ and K_4_Fe(CN)_6_—meets the experimental requirements. The theoretical value of the peak potential difference is 64 mV, and a peak separation in the range of 80–90 mV is generally considered acceptable for practical use. The theoretical formula for calculating the peak potential difference is given in Equation (1). By substituting the relevant values into the equation, the value of ΔΦ can be determined.

(1) $$\Delta \;E=\mathrm{Epa}-\mathrm{Epc}=2.2\frac{RT}{nF}=\frac{58}{n}$$

In the formula: Epa and Epc are the anodic (oxidation) and cathodic (reduction) peak potentials, respectively; *R* is the gas constant; *T* is the temperature; *F* is the Faraday constant; and *n* is the number of electrons transferred.

**Figure 8:**
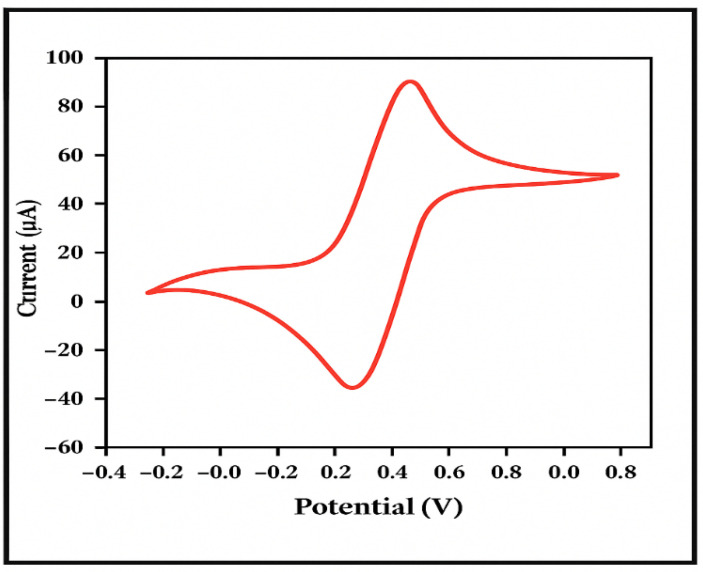
Cyclic voltammogram of GCE after polishing and cleaning in a 5 mM K_3_Fe(CN)_6_/K_4_Fe(CN)_6_ solution.

Mechanically polish the GCE sequentially using alumina powder with particle sizes of 1.0, 0.3, and 0.05 µm until a mirror-like surface is obtained. Rinse the surface of the GCE with ethanol and deionized water, then clean it in an ultrasonic bath and dry it with nitrogen gas to obtain a suitable glassy carbon electrode for use. Set the voltage range for the cyclic voltammetry (CV) test from −0.2 to 0.7 V. Perform the CV test of the polished GCE in an aqueous solution containing 5 mM K_3_Fe(CN)_6_ and K_4_Fe(CN)_6_, as shown in Figure 8. In the resulting CV curve, redox peaks with nearly equal current values are observed. The anodic peak potential is approximately 270 mV, and the cathodic peak potential is approximately 195 mV. The peak potential difference is 75 mV, which falls within the theoretical range, indicating that the treated GCE is suitable for testing.

Cyclic voltammogram of GCE after polishing and cleaning in a 5 mM K_3_Fe(CN)_6_/K_4_Fe(CN)_6_ solution (Figure 8). The red curve represents the cyclic voltammetric response of the polished and cleaned GCE, and the color is used solely for visual distinction of the recorded CV signal.

### 5.4. Voltammetric Testing of Pb^2+^ and Cd^2+^ Stripping

Electrochemical testing using differential pulse voltammetry (DPV) combined with in situ deposition of a bismuth film was employed in the experiment. The testing of Pb^2+^ was carried out as follows: (1) Blank sample determination: prepare 15 mL of an acetate buffer solution containing 100 μg/L Bi^3+^. Connect the solution to a three-electrode electrochemical testing system, apply a deposition potential of −1.2 V at a stirring rate of 500 rpm, and perform preconcentration for 120 s. Then, under quiescent conditions, conduct stripping in the potential range of −1.2 to 0.4 V. After stripping, apply a constant potential of 0.3 V for electrochemical cleaning of the electrode.

(2) Determination of 2 μg/L Pb^2+^: after the blank sample test, add 15 μL of Pb^2+^ solution with a concentration of 2 μg/mL into the solution, so that the Pb^2+^ concentration remains at 2 μg/L. Then repeat the preconcentration and stripping process. After each stripping, perform electrochemical cleaning of the electrode at a constant potential of 0.3 V. Each sample is tested 7 times, and the stripping potential and stripping current are recorded.

(3) Determination of 10 μg/L Pb^2+^: to the above solution, 60 μL of Pb^2+^ solution with a concentration of 2 μg/mL was added, so that the Pb^2+^ concentration in the solution reached 10 μg/L. The preconcentration and stripping process was then repeated. After each stripping, the electrodes were subjected to electrochemical cleaning at a constant potential of 0.3 V. Each sample was tested seven times, and the stripping potential and stripping current were recorded.

### 5.5. MXene-Anode-Glucose Oxidase/Prussian Blue/ITO-Cathode Self-Powered System for the Determination of Mercury (II) in Water

The mercury ion (Hg^2+^) is one of the most stable inorganic forms of mercury and poses a serious threat to environmental safety and human health. Considering its danger to the environment and living organisms, researchers are continuously working on the development of sensitive, rapid, and cost-effective methods for the detection of Hg^2+^. At present, various sensing systems are used for the accurate analysis of mercury in environmental samples, such as atomic absorption spectroscopy, atomic fluorescence spectroscopy, inductively coupled plasma mass spectrometry (ICP-MS), and atomic emission spectroscopy. However, these methods have several drawbacks, including operational complexity, high equipment cost, and the requirement for skilled personnel. In recent years, electrochemical technologies have attracted significant attention in the field of Hg^2+^ detection due to their high selectivity, low cost, and ease of miniaturization. To meet the growing demand for inexpensive, rapid, reliable, and sensitive methods for heavy metal detection, extensive research has been carried out on the development of low-cost and user-friendly detection systems for heavy metals.

Traditional electrochemical sensors usually consist of four parts: a recognition element, a signal conditioning and conversion circuit, a signal amplifier, and an auxiliary power supply circuit [125]. In contrast, self-powered electrochemical sensors (SPES) do not require batteries or an external power source, as they harvest energy from the environment through electrochemical processes, thereby providing self-sustained operation of the sensor [126]. By utilizing mediated or direct electron transfer between enzymes and electrodes, efficient charge transfer across the interface can be achieved [127,128]. In recent years, enzyme-based SPES have attracted considerable attention. Enzymes operate under mild conditions and can maintain high activity at reasonable ambient temperatures and neutral pH [129,130]. A self-powered sensor consists of two electrodes, with the sensor itself supplying power to the sensing device. The simple two-electrode design and the ability to function without an external power source not only provide SPES with enhanced stability and simplified operation but also allow for easy miniaturization and simplified manufacturing. This eliminates the need for an external power supply and addresses the limitation hindering miniaturization, making it an effective strategy for developing SPES.

In addition to its excellent biocompatibility, outstanding electrical conductivity, large specific surface area, and other properties, studies have shown that MXene is highly susceptible to oxidation when exposed to air. The ease of oxidation of MXene, together with its large specific surface area, demonstrates its potential as an electron donor. In this study, the focus is placed on the redox properties of MXene, which, combined with the reversible transformation between Prussian blue (PB) and Prussian white (PW), enables the construction of a self-powered system consisting of an MXene anode–glucose oxidase (GOD)/PB/ITO cathode for the detection of Hg^2+^ in water [131].

## 6. Results and Discussion

### 6.1. Determination of Trace Lead (II) and Cadmium (II) in Water Using a Glassy Carbon Electrode

The limit of detection (LOD) refers to the minimum amount of a measurable component that can be detected in a sample. The method detection limit (MDL) is the minimum concentration or the smallest amount of a measurable substance that can be qualitatively detected in a sample with a given level of confidence using a specific analytical method. To determine the MDL, the sample must be processed through all the steps of the specified analytical method. It is one of the key parameters characterizing an analytical method. Lead and cadmium, as common sources of heavy metal pollution, can have immeasurable toxic effects on the human body, posing a serious threat to human health. Lead and cadmium are mainly present in water in the form of ions (Pb^2+^ and Cd^2+^). Conventional detection methods such as spectroscopy, chromatography, and spectrophotometry have high usage thresholds, are complex to operate, and are not suitable for the rapid and simple detection of heavy metals. Differential pulse voltammetry (DPV) combines preconcentration, dissolution, and electrochemical measurement of heavy metal ions, making it an extremely sensitive and rapid electroanalytical method.

**Figure 9:**
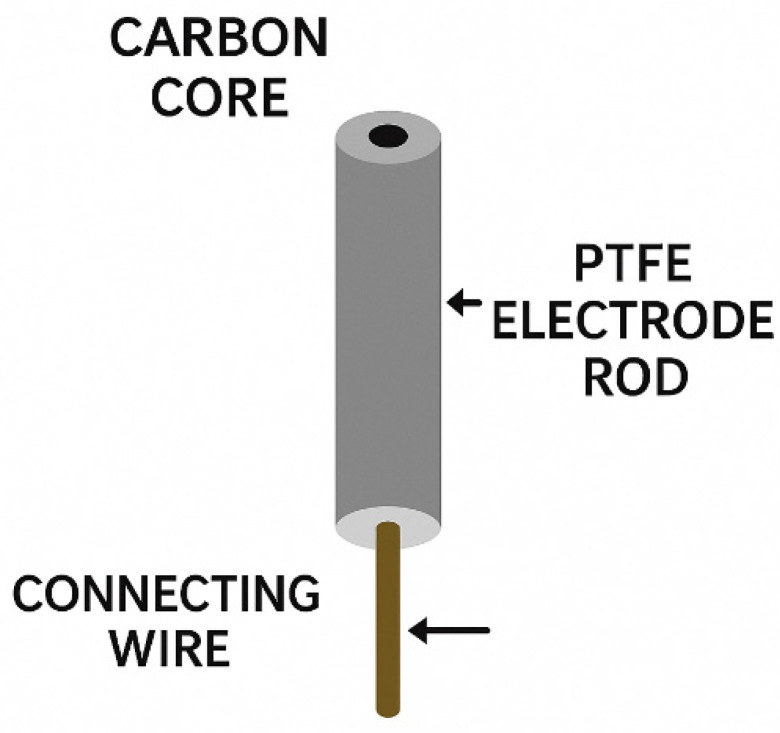
Schematic representation of a glassy carbon electrode (GCE).

The glassy carbon electrode (GCE) is a widely used electrode in electrochemical experiments, as shown in Figure 9. Due to its excellent electrical conductivity, outstanding chemical stability, robust and durable structure, low thermal expansion coefficient, high sealing performance, and wide electrochemical potential window, it is extensively used in electrochemical experiments.

Zhang et al. [109] used a GCE modified with carbon dots (CDs) and a Nafion-modified bismuth film to detect Cd^2+^ and Pb^2+^ in water. In the experiment, the LOD for Pb^2+^ and Cd^2+^ was found to be 2.3 µg/L and 3.1 µg/L, respectively. Sereilakhena Phal et al. [110] used a GCE modified with Bi/carboxyphenyl for the detection of Pb^2+^ and Cd^2+^ in water. The LOD for Pb^2+^ and Cd^2+^ in the experiment was 10 µg/L and 25 µg/L, respectively.

Amine Djebbi et al. [132,133] used a GCE modified with biomass-derived porous carbon (BPC) and nanoscale zero-valent iron (nZVI) for the detection of Pb^2+^ in water. The experimentally determined LOD for Pb^2+^ was 2.2 µg/L. The aforementioned detection methods require complex modification of the GCE. In this study, a bare GCE was used as the working electrode, simplifying the GCE modification process. A silver/silver chloride (Ag/AgCl) electrode was used as the reference electrode, and a platinum wire as the counter electrode. Using differential pulse voltammetry (DPV), the LOD and MDL of Pb^2+^ and Cd^2+^ in water were determined, enabling sensitive detection of Pb^2+^ and Cd^2+^.

### 6.2. Analytical Performance of the MXene–GOD/PB/ITO Self-Powered Sensor for Hg^2+^ Detection

The analytical performance of the MXene–GOD/PB/ITO self-powered sensor for Hg^2+^ detection was evaluated in terms of linear detection range, limit of detection (LOD), sensitivity, selectivity, repeatability, and operational stability. Owing to the excellent electrical conductivity of MXene and the efficient biocatalytic activity of glucose oxidase (GOD), the constructed self-powered system demonstrated highly sensitive electrochemical response toward Hg^2+^ ions.

Under optimized conditions, the sensor exhibited a linear response to Hg^2+^ concentration in the range of 1–5 μg/L, indicating its suitability for trace-level mercury detection in environmental water samples. The calculated limit of detection (LOD) reached 50 ng/L, which is significantly lower than the maximum allowable concentration of mercury in drinking water set by the World Health Organization (WHO). This low detection limit confirms the high analytical sensitivity of the proposed self-powered sensing platform.

The sensing mechanism is based on the inhibitory effect of Hg^2+^ on the enzymatic activity of glucose oxidase. In the absence of Hg^2+^, GOD catalyzes the oxidation of glucose to generate hydrogen peroxide (H_2_O_2_), which subsequently oxidizes Prussian White (PW) back to Prussian Blue (PB), resulting in a distinct color change and a measurable electrochemical signal. In the presence of Hg^2+^ ions, the activity of GOD is suppressed due to strong Hg^2+^–enzyme coordination, leading to reduced H_2_O_2_ production and weaker PB/PW conversion. As a result, the output signal intensity decreases proportionally with increasing Hg^2+^ concentration. This inhibition-based sensing strategy ensures high selectivity toward Hg^2+^ over other coexisting metal ions.

Selectivity studies showed that common interfering ions such as Pb^2+^, Cd^2+^, Zn^2+^, Cu^2+^, and Fe^3+^ caused negligible signal interference, confirming the excellent anti-interference capability of the MXene–GOD/PB/ITO sensor. The repeatability of the sensor was evaluated through multiple successive measurements, yielding a relative standard deviation (RSD) of less than 6%, indicating good operational reproducibility. Furthermore, the sensor retained over 90% of its initial response after two weeks of storage, demonstrating acceptable long-term stability.

Compared with previously reported Hg^2+^ detection systems, such as microbial fuel cell (MFC)-based biosensors and modified carbon electrodes, the MXene-based self-powered sensor developed in this work exhibits a lower detection limit, simpler device structure, and does not require an external power source. Scientists reported an MFC-based Hg^2+^ biosensor with a detection range of 0.2–3 mg/L and a detection limit of 50 μg/L, which is significantly higher than the value achieved in this study. Therefore, the present MXene–GOD/PB/ITO self-powered system demonstrates superior analytical performance for trace mercury detection in aqueous environments.

Moreover, the integration of MXene as a non-enzymatic anode material significantly enhances electron transfer efficiency due to its large specific surface area and rich surface functional groups. This feature promotes rapid charge transport at the electrode–electrolyte interface and improves the overall sensitivity of the self-powered sensor. These advantages are consistent with previously reported MXene-based electrochemical sensing platforms for heavy metal detection [134,135].

Overall, the excellent analytical performance, low detection limit, high selectivity, good repeatability, and operational stability indicate that the MXene–GOD/PB/ITO self-powered sensor is a promising portable platform for on-site Hg^2+^ monitoring in environmental water samples.

Recent advances further confirm the central role of carbon-based and hybrid materials in electrochemical sensing. Recent reviews on portable and screen-printed electrochemical sensors highlight their growing importance for on-site determination of heavy metal ions in environmental waters, as these platforms combine low cost, portability, and reliable analytical performance for Pb^2+^ and Cd^2+^ detection under field conditions [136].

Although MXenes are not strictly classified as carbon-based materials, they are frequently integrated with carbon electrodes and carbon nanostructures to form hybrid electrochemical sensing platforms. Their outstanding electrical conductivity, hydrophilicity, and strong interfacial interaction with carbon substrates enable highly efficient charge transfer and enhanced signal stability. Therefore, MXene-based systems are discussed in this review as carbon-related hybrid electrochemical sensors.

## 7. Conclusions

The obtained LOD and MDL values were also compared with international regulatory limits. According to the World Health Organization (WHO), the permissible limits for Pb^2+^ and Cd^2+^ in drinking water are 10 µg/L and 3 µg/L, respectively, while the United States EPA sets limits of 15 µg/L for Pb^2+^ and 5 µg/L for Cd^2+^. The LOD values achieved in this study (0.405 µg/L for Pb^2+^ and 0.565 µg/L for Cd^2+^) are significantly lower than both WHO and EPA thresholds, confirming that the developed sensing systems are suitable for detecting trace concentrations well below regulatory requirements. For Hg^2+^, the achieved linear range of 1–5 µg/L aligns with the WHO guideline value of 6 µg/L, demonstrating that the self-powered MXene-based sensor can reliably detect mercury at environmentally relevant levels.

In addition to meeting regulatory criteria, the sensors demonstrate strong practical advantages: low cost, portability, and rapid response without the need for complex surface modifications. The excellent performance of the bare GCE can be attributed to the efficient electron-transfer kinetics, its wide potential window, and the ability of the carbon surface to promote diffusion-controlled preconcentration of metal ions. The absence of surface modifiers also minimizes variability between electrodes, contributing to improved reproducibility. These characteristics explain why even the unmodified GCE achieved very low detection limits for Pb^2+^ and Cd^2+^.

These findings highlight that the developed carbon-based and MXene-assisted sensing strategies not only achieve high analytical sensitivity but also hold strong potential for practical field applications, especially in rapid and on-site environmental monitoring.

## Figures and Tables

**Table 1:** Summary of LOD and MDL Values.

Metal	LOD (µg/L)	MDL (µg/L)	WHO Limit (µg/L)	EPA Limit (µg/L)
Pb^2+^	0.405	0.424	10	15
Cd^2+^	0.565	0.592	3	5
Hg^2+^	1–5	—	1	2

**Table 2:** Comparison with Literature.

Electrode	Technique	Metal	LOD	Reference
Bare GCE	DPV	Pb^2+^	0.405 µg/L	This work
Bare GCE	DPV	Cd^2+^	0.565 µg/L	This work
MXene/GOD-PB/ITO	Self-powered	Hg^2+^	1–5 µg/L	This work
Sb electrode	PSA	Pb^2+^	0.03 µg/L	Wei et al. [51]

**Table 3:** Key Electrochemical Parameters.

Metal	Linear Range	Sensitivity	Repeatability (RSD %)	Stability
Pb^2+^	µg/L range	High	≤5%	Good
Cd^2+^	µg/L range	High	≤6%	Good
Hg^2+^	1–5 µg/L	Moderate	≤7%	Good

## Data Availability

All data supporting the findings of this study are included within the article.
